# Family burden in inherited ichthyosis: creation of a specific questionnaire

**DOI:** 10.1186/1750-1172-8-28

**Published:** 2013-02-15

**Authors:** Hélène Dufresne, Smail Hadj-Rabia, Cécile Méni, Vincent Sibaud, Christine Bodemer, Charles Taïeb

**Affiliations:** 1 Department of Dermatology, Necker-Enfants Malades Hospital, Centre de Référence National pour les Maladies Génétiques à Expression Cutanée (MAGEC), APHP, Paris, France; 2 Université Paris V-Descartes, Paris, France; 3 Eau Thermale Avène, Lavaur, France; 4 Public Health and Quality of Life, Pierre Fabre, Boulogne, France

**Keywords:** Global burden disease, Quality of life, Ichthyosis, Burden questionnaire

## Abstract

**Background:**

The concept of individual burden, associated with disease, has been introduced recently to determine the “disability” caused by the pathology in the broadest sense of the word (psychological, social, economic, physical). Inherited ichthyosis belong to a large heterogeneous group of Mendelian Disorders of Cornification. Skin symptoms have a major impact on patients’ Quality of Life but little is known about the burden of the disease on the families of patients.

**Objectives:**

To develop and validate a specific burden questionnaire for the families of patients affected by ichthyosis.

**Methods:**

Two steps were required. First, the creation of the questionnaire which followed a strict methodological process involving a multidisciplinary team and families. Secondarily, the validation of the questionnaire, including the assessment of its reliability, external validity, reproducibility and sensitivity, was carried out on a population of patients affected by autosomal recessive congenital ichthyosis. A population of parents of patients affected by ichthyosis was enrolled to answer the new questionnaire in association with the Short Form Q12 questionnaire (SF-12) and a clinical severity score was filled for each patient.

**Results:**

Ninety four families were interviewed to construct the verbatim in order to create the questionnaire and a cognitive debriefing was realized. The concept of burden could be structured around five components: “economic”, “daily life”, “familial and personal relationship”, “work”, and “psychological impact”. As a result, “Family Burden Ichthyosis” (FBI) reproducible questionnaire of 25 items was created.

Forty two questionnaires were analyzable for psychometric validation. Reliability (Cronbach’s alpha coefficient = 0.89), reflected the good homogeneity of the questionnaire. The correlation between mental dimensions of the SF-12 and the FBI questionnaire was statistically significant which confirmed the external validity. The mean FBI score was 71.7 ± 18.8 and a significant difference in the FBI score was shown between two groups of severity underlining a good sensitivity of the questionnaire.

**Conclusions:**

The internal and external validity of the “FBI” questionnaire was confirmed and it is correlated to the severity of ichtyosis. Ichthyoses, and other chronic pathologies, are difficult to assess by clinical or Quality of Life aspects alone as their impact can be multidimensional. “FBI” takes them all into consideration in order to explain every angle of the handicap generated.

## Background

Inherited ichthyoses form part of a large, clinically and etiologically heterogeneous group of Mendelian Disorders of Cornification and typically involve all or most of the tegument [1]. All are characterized by chronic dryness, hyperkeratosis and scaliness [1-3]. Both syndromic (with extra-cutaneous manifestations), and non syndromic forms of ichthyoses are described [1,4]. In non syndromic inherited forms, the main types are the ichthyosis vulgaris (MIM#146700) [5], the X-linked recessive ichthyosis (MIM#308100) and Lamellar Ichthyosis (LI; prevalence < 1:100000) which is one of the Autosomal Recessive Congenital Ichthyosis (ARCI) [1,3,4]. In the recent new classification, ARCI refers to Harlequin Ichthyosis and LI/Congenital Ichtyosiform Erythroderma (CIE) phenotypic spectrum of disorders [1]. LI is characterized by coarse and brown or dark scaling [5]. Neonates with Harlequin Ichthyosis go on to express a severe LI-like phenotype which evovlves into generalized exfoliating erythrodermic ichthyosis, whereas CIE is characterized by fine and white scaling with varying degrees of erythema. In fact, ARCI is characterized by visible signs and distressing symptoms such as pruritus, fissures and cracks, limited joints movements, reduced cutaneous sensitivity, water loss, infectious risks, and sometimes severe ectropion and/or eclabion. Global management is symptomatic and often time-consuming. It includes baths, topical emollient, topical keratolytic agents and sometimes oral retinoid [3,4,6-9].

Several studies have focused on the negative impact of ichthyosis on Quality of Life (QoL) mostly because of severe erythema and hyperkeratoses [3,5,10-12]. However, a specific questionnaire with a precise evaluation of the impact of ichthyoses on the different aspects of daily life has never been proposed.

Furthermore, for several years, the concept of “burden” has taken an increasingly important place in the medical field in evaluating the care of chronic diseases. It was introduced by the World Health Organization and was particularly useful for quantifying the health of a population and determining the priorities of action in the public health domain [13]. More recently, the concept of individual disease burden has been introduced to determine the “disability” in the broadest sense of the word (psychological, social, economic, physical), and to distinguish it from societal burden, which is primarily concerned with its economic impact [14,15].

To assess the burden, data are collected using questionnaires and there is a well defined methodology to built and validate QoL questionnaires. However, the burden questionnaires are still poorly developed. Two previous published questionnaires for evaluating the burden were established according to this rigorous methodology of QoL questionnaires: the “Burden of fibromyalgia” and the “Burden of chronic venous disorders” [14,16].

The aim of this study was to create and validate a French questionnaire to evaluate the burden on families of patients affected by ichthyosis.

## Patients and methods

**Figure 1:**
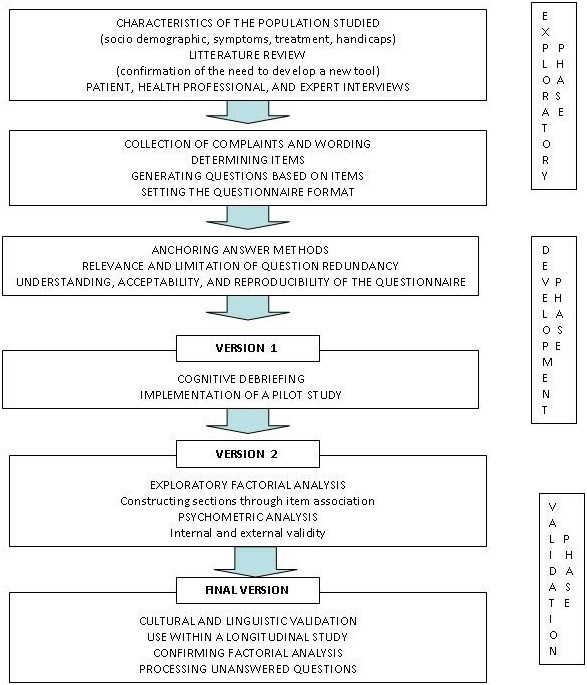
Stages in questionnaire development.

We decided to develop a specific Family Burden Ichthyosis questionnaire (“FBI”) according to the rigorous methodology of construction of QoL questionnaires (Figure 1) by two steps: creation of the questionnaire and validation [17-19]. This methodology required a multidisciplinary team involved in the care of the patients and their families.

### Creation of the questionnaire

#### The study population: the “verbatim population”

Between July 2005 and December 2010, all the daily life data concerning the ARCI patients followed in the “Centre de Référence National des Maladies Génétiques à Expression Cutanée” (MAGEC) and their parents, and the patient's and family's complaints, were systematically collected by the social worker. These data were used for the creation of a verbatim. The diagnosis of ARCI was based on clinical, histological and when available, molecular results.

#### Construction process

This first step can be divided into three stages.

##### Stage 1: creation of the verbatim

Data collection including the different complaints expressed by parents and by affected children themselves during a one-on-one session with the same social worker. For that, a French social assessment has been used, inspired by a standardized methodology (available on request).

##### Stage 2: analysis of the “verbatim”

This stage allowed the creation of a specific and relevant questionnaire. The analysis has been realized by the social worker and by the physicians. All questions have been gathered, and submitted to a specialized team in the management of ichthyosis at MAGEC. The aim was to simplify the questionnaire and to avoid redundancies. The questionnaire was created in a question and answer format.

##### Stage 3

The cognitive debriefing was managed by the Lionbridge Company. The aim was to verify the comprehension of the original French questionnaire in terms of use of words and use of vocabulary to permit a good understanding by everyone (i.e. different socio-professional categories, duration of the disease; [20]). A French native with a strong background in cognitive interviewing techniques conducted each interview. The questionnaire had to be discussed and modified if necessary. The samples had to be sufficiently representative of the population for which the instrument was designed and the questionnaire was written in their mother language.

### Validation of the questionnaire

#### Study population: the “validation” population

The subjects fulfilling the following criteria were included in the burden evaluation: parents of an affected child (age < 18 years-old) suffering from ARCI follow in MAGEC, fluent in French language, with oral consent for participation. Inclusion started in May 2011 and was stopped when at least thirty subjects were enrolled. This number was validated in previous epidemiologic studies [21].

#### Validation process

Validation of the questionnaire included assessing its reliability, validity, reproducibility and sensitivity. The questionnaire was anonymously administered to a group of parents (mother or father). As it was anonymous, an approval of an ethics committee was not considered as necessary by our administrative instances.

The questionnaire distributed to the families consisted of the new specific questionnaire to be validated, a common questionnaires: the Short-Form Q-12 (SF-12) and a scale to evaluate the severity of ichthyosis [5]. The SF-12 is a common, validated QoL questionnaire that is simple and short [22]. It measures the QoL based on the overall health status. It has 2 dimensions: physical (PCS) and mental (MCS), with scores between 0 and 100. The higher the score, the better the QoL. The SF12 is validated in French. The severity of ichthyosis for each child, was assessed with the published score of severity of MAGEC [5]. It is based on the sum of the percentage of the scaly surface, the percentage of the erythematous surface, the intensity of pruritus and skin pain as measured through visual analogue scale, and the severity of 10 symptoms of ichthyosis (determined on a scale of 60).

The reliability or internal consistency was measured by Cronbach’s α coefficient. This coefficient including 0 to 1 corresponds to a degree of homogeneity (internal consistency). Coefficient scores > 0.7 usually indicate good internal reliability or internal consistency. Furthermore, to evaluate the external validity of the questionnaire and the equability of the scale of our FBI questionnaire a comparison was performed with the SF-12 [23]. The cognitive debriefing stage can replace with a relevant and efficient method the test-retest step to study the reproducibility (fewer patients involved, faster and more professional). Finally, the sensitivity was assessed by comparing the FBI score and the severity score.

The linguistic, cultural adaptation and validation have subsequently made it possible for the “FBI” to be available in English, based on the guidance of the International Society for Pharmaco-economics and Outcomes Research Principles of Good Practice for Translation [24]. The final questionnaire was then tested in a sample of native English UK-speaking subjects during an individual, cognitive debriefing interview to determine the issues related to question and answer wording (ambiguity, misunderstanding, acceptability, etc.). This cultural adaptation and validation was performed by a specialized institution (Lionbridge, Ireland).

### Statistical analysis

The quantitative variables were compared using Student's *t*-test or an ANOVA when there were more than two groups. When the conditions necessary for the application of these tests were not met, non-parametric tests were used to compare the two groups (Mann Whitney Wilcoxon). The qualitative variables were compared using a Χ^2^ test. When the conditions necessary for the application of this test were not met, the Fisher exact test was preferred. A Cronbach’s α coefficient was measured to evaluate the reliability and a Spearman’s correlation coefficient (r) was calculated to assess the validity.

## Results

### Creation of the questionnaire

#### Construction process

The different steps showed that the concept of “burden” could be structured around five dimensions: pain, daily life, familial and personal relationship, work, and psychological impact. The main topics and complaints expressed by parents, found in the verbatim, were: “the difficulties in finding someone to babysit their children”, “the organization of holidays”, “the restrictions in terms of leisure”, “the society’s perception”, and “the couple’s problems”. The complaints least expressed by parents were: “the management of siblings”, “the administrative records to file”, “the itching at night”, and “the alimentation”. By using the verbatim, ninety six questions were identified. However, it was decided that the expression “the skin disease of our children” have to be included in each item, to highlight the relation between skin disease and each dimension.

The secondary analysis of the ninety six first questions allowed a reduction to forty items. For example, three questions about the general impact on the family QoL were available: “the skin disease of our child makes our life difficult”, “the skin disease of our child disrupts our family life”, “the skin disease of our child disturbed our family life”, finally one question was chosen: “the skin disease of our child complicates our family life”. The item “I feel guilty because of the skin disease of our child” was modified to “I feel responsible for the skin disease of our child” making it more relevant. At the same time, we retained the different dimensions, while improving their use.

The cognitive debriefing was realized and some questions were changed to be more clear or easier to understand by reforming sentences through changing its order of words. For example, the item “During the day, I think about my child’s skin disease continually” was changed to “I spend the day thinking about my child's skin disease”.

Finally, a multidimensional questionnaire: the “Family Burden Ichthyosis questionnaire” was available in French for parents which is simple to use and easy to understand, consisting of forty items, and could be considered as the final version to be validated. Each response was scored from 0 to 3 (Table 1).

**Table 1:** Below are a series of statements concerning your child’s skin disease, please answer as spontaneously as possible

My child's skin disease has made us want to move.	□definitely yes	□maybe	□definitely not	□I don’t know
My child's skin disease has made me want to quit my job.	□definitely yes	□maybe	□definitely not	□I don’t know
My child's skin disease affects my sleep.	□definitely yes	□maybe	□definitely not	□I don’t know
My child's skin disease complicates our family life.	□definitely yes	□maybe	□definitely not	□I don’t know
I spend the day thinking about my child’s skin disease	□definitely yes	□maybe	□definitely not	□I don’t know
My child’s skin disease prevents us from going on vacation.	□definitely yes	□maybe	□definitely not	□I don’t know
My child needs more attention than other children because of his/her skin disease.	□definitely yes	□maybe	□definitely not	□I don’t know
Our child’s skin disease has forced us to rethink our plans for the future.	□definitely yes	□maybe	□definitely not	□I don’t know
Our child's skin disease causes us to neglect his/her brothers and sisters.	□definitely yes	□maybe	□definitely not	□I don’t know
My child’s skin disease prevents me from going to see my family.	□definitely yes	□maybe	□definitely not	□I don’t know
Because of my child’s skin disease, my family does not come to see us.	□definitely yes	□maybe	□definitely not	□I don’t know
Our child’s skin disease creates problems between me and my partner.	□definitely yes	□maybe	□definitely not	□I don’t know
I often feel frustrated after consultations related to our child’s skin disease.	□definitely yes	□maybe	□definitely not	□I don’t know
People's reactions to our child's skin disease are hard to accept.	□definitely yes	□maybe	□definitely not	□I don’t know
I feel guilty because of our child's skin disease.	□definitely yes	□maybe	□definitely not	□I don’t know
My child's skin disease has completely disrupted my life.	□definitely yes	□maybe	□definitely not	□I don’t know
I have not managed to accept our child's skin disease.	□definitely yes	□maybe	□definitely not	□I don’t know
I have a hard time getting used to the smell caused by our child's skin disease.	□definitely yes	□maybe	□definitely not	□I don’t know
Because of his/her skin disease, I have great difficulty finding someone to babysit my child.	□definitely yes	□maybe	□definitely not	□I don’t know
Because of his/her skin disease, my child has a lot of difficulties at school.	□definitely yes	□maybe	□definitely not	□I don’t know
Because of his/her skin disease, I fear for the future of my child.	□definitely yes	□maybe	□definitely not	□I don’t know
I'm growing tired of the daily care.	□definitely yes	□maybe	□definitely not	□I don’t know
I don’t feel well the day before I go to hospital.	□definitely yes	□maybe	□definitely not	□I don’t know
I don’t feel well the day after I go to hospital.	□definitely yes	□maybe	□definitely not	□I don’t know
The care I must provide to my child is extremely tiring.	□definitely yes	□maybe	□definitely not	□I don’t know

There are no rights or wrongs answers, only your answer. Thank you for your participation.

### Validation of the questionnaire

#### Population of the study: the “burden’s evaluation” population

The questionnaires were administered to the parents of 42 independent patients (17 girls, 25 boys mean age of 7.9 ± 4, 1; Table 2) suffering from LI or CIE from May to August 2011. No one of these patients took part in the verbatim group.

**Table 2:** Validation population

	**Lamellar ichthyosis**	**CIE**
**Patients**	**30**	**12**
**Gender (F/M)**	**12/18**	**5/7**
**Age**	**8.3 (0–16.5)**	**7.6 (0–15)**
**Collodion Baby Syndrome**	**24**	**0**
**Oral Acitretin**[1]	**0**	**5**

*CIE* = Congenital Ichtyosiform Erythroderma, *F* = Female, *M* = Male. [1] Topical tazaroten and urea were not mentioned but have been used sporadically.

### Validation process

#### Reproducibility

Reproducibility of the questionnaire was realized with the cognitive debriefing method, and led us to modify the structure of some questions.

#### Reliability

The Cronbach α coefficient is 0.89, signifying a very good internal consistency of the scale and a good homogeneity of the forty items.

#### Validity

The scale was compared to SF-12. The two SF-12 dimensions (mental and physical) were calculated. The mean score for the SF-12 mental dimension was 28.7 ± 7.96 which expressed a significant impairment of the QoL. The mean score for the physical dimension was 51.3 ± 6.9, which didn’t express an impairment of QoL. The FBI overall score, transformed into a scale of 100 points, was calculated. The mean score was 71.71 ± 18.8, and the scores of each dimension were also transformed to a 100-point scale. The "daily’life" and "psychological impact" dimensions were the most affected by the disease. The correlations between FBI, severity score and SF-12 scores are reported in Table 3. The correlation is statistically significant for the mental dimensions of the SF-12 and the FBI (α = -0.564, p = 0.002), but not for the physical dimension (α = -0.012, p = 0.46).

**Table 3:** Correlation between severity and FBI (dimension by dimension), SF-12 and FBI

	**“Familial and personal relationship”**	**“Daily life”**	**“Economic”**	**“Work”**	**“Psychological impact”**	**Severity score**	**SF12 Physical dimension**	**SF12 Mental dimension**
FBI						0.73283	−0.12121	−0.56438
						<.0001	0.4623	0.0002
						**		**
**SF12 Physical dimensions**	−0.27727	−0.14158	−0.14644	−0.03406	−0.05190	−0.08413		
	0.0875	0.3899	0.3737	0.8369	0.7537	0.6106		
**SF12 Mental dimensions**	−0.51752	−0.45132	−0.50177	−0.57600	−0.50151	−0.50815		
	0.0007	0.0039	0.0011	0.0001	0.0011	0.0010		
	**	**	**	**	**	**		
**Severity Score**	0.61204	0.67932	0.72605	0.54549	0.49912			
	<.0001	<0.0001	<.0001	0.0002	0.0008			
	**	**	**	**	**			
**Cronbach α coefficient**	0.82	0.71	0.84	0.72	0.64			

#### Sensitivity of FBI and severity scores

The mean severity score was 45.89 ± 22.86 (with a minimum of 5 and a maximum of 94), and 17 subjects had a score ≥ 50 (40.40%). The mean severity score for girls was 43.37 ± 22.83 and for boys 48.54 ± 24.92. The difference between the two groups was not significant (p = 0.24). Furthermore, the severity score in children aged under seven years was 42.68 ± 22.94 and, in children aged from seven years or more, was 48.48 ± 23.71, the different was also not significant (p = 0.20). The population was homogeneous enough, it was not necessary to do subgroups with age and sex. However, we realized subgroups of severity; one group with a specific severity score strictly under 50 (subgroup 1) and the other group with a specific severity score greater than or equal to 50 (subgroup 2). In the subgroup 1 the overall FBI score was 60.72 ± 19.16. In the subgroup 2 the overall FBI score was 85.01 ± 7.23. The difference between the two groups was statistically significant (p <0.0001). The scores of the five dimensions (economic, daily life, familial and personal relationship, work, and psychological impact) were statistically different between these two subgroups. The p-values were 0,00001, 0,00002, 0,00004, 0,00109 and 0,00016 respectively. The more important the severity was, the more altered the scores were (Table 3).

## Discussion

Ichthyoses constitute a group of rare, chronic, and debilitating diseases that are difficult to assess solely by clinical or QoL elements, as their impact can be multidimensional [3,5,10-12]. The DLQI scores place ichthyoses among the skin disorders with the most harmful impact on a patient’s QoL [5]. In children, the dermatologist does not limit action to the skin care [6,9] but also aims to prevent the sensory and psychomotor consequences of these conditions [2,4,5]. Of note, it was also revealing an increase of the resources utilization and cost (in the United States) [10,25]. Thus, a global evaluation instrument is needed (Figure 1). The provision of a specific evaluation tool for assessing the burden of ichthyoses to healthcare professionals is necessary for an objective and constructive evaluation. Global burden is considered as a “health breach” and allows the health authorities to plan some pertinent health programs. Otherwise the individual burden takes care of the patient himself (or its family, its care giver) and describes the disability produced by the disease in the broadest sense of the word (psychological, social, economic, physical). It allows to adapt the management of patients in order to objectify and optimize the improvement of their health [14,16]. The benefit of our questionnaire is that it evaluates the family’s feelings through the burden that conditions and takes into account the QoL, integration within the community, life organization and the level of medical resources consumed (medical visits, treatment, etc.). It is also possible to assess a drug or a non medicinal management with the modification of the burden.

This questionnaire, the “FBI”, is the first powerful specific questionnaire compiled for ichthyosis in French and was validated during the study (Table 1). It takes each of the dimensions (pain, daily life, familial and personal relationships, work, and psychological impact) into account to express the burden produced by this disease. The validity of the FBI is confirmed by the significant correlation with the score of the mental dimension of the SF-12 (-0.564, p = 0.0002). Otherwise the score of the physical dimension of the SF-12 didn’t suggest an impairment of QoL and the correlation with the physical dimension was not significant (Table 3). It could be explained because the FBI is a burden questionnaire for families and not for children, it was not surprising that the physical dimension assessed in parents was not altered. The relative young age of parents could be another explanation. Other studies assessing the QoL of parents of children affected by cutaneous diseases using the SF-12 to compare with a more specific questionnaire found the same results about the physical dimension of SF-12 [26].

Even when the sample of patients is not very important, we compared the FBI score with the severity score assessed by the specific instrument create by MAGEC, in order to assess the sensitivity of the FBI. The population was organized into two subgroups according to the specific severity score being above or below fifty. The overall score of the FBI proved to be highly and statistically correlated with the severity score. The five FBI dimensions were significantly correlated with the severity score. Furthermore, it was shown that the more important the severity was, the more altered the scores were. These results underlined a good sensitivity of the new questionnaire. This fact implies that globally, the FBI manages to show the overall important impact of ichthyosis in all parents of patients with this chronic and outwardly physical condition that should not be underestimated, even in the less severe clinical forms.

These first results of FBI score, in a group of 42 ARCI parents showed a high score and then, an important impact on daily’s life of families (Tables 2 and 3).

Although the study is monocentric, it does not constitute a limit of the study. Indeed, the studied population is representative because MAGEC follows patients living in France (and not only in Paris). On the other hand, there is a selection’s bias given that the patients followed in the center are affected by severe forms (severity score greater or equal to fifty for 17 patients).

The FBI was also translated into English according to the good practices based on cross cultural validation (Table 1). The cross cultural validation will be realized in German, Italian and Spanish. The next step in the process of assessing burden of families with children affected by ichthyosis will be to evaluate the possible changes in the severity of the disease burden before and after treatment. More specific aspects of other ichthyoses (i.e. Netherton Syndrome and keratinopathic ichthyosis) need to be discussed.

## Conclusion

Our FBI questionnaire seemed to be a good tool for evaluating the burden on families of patients with ichthyosis, and to be useful for improved multidisciplinary monitoring of these patients and their family. With this “Family Burden Ichthyosis” questionnaire, all aspects of the multidimensional impact will be taken into consideration in order to explain every angle of the handicap generated contrary to a QoL questionnaire.

Additionally, the FBI questionnaire will help to validate different strategies to reduce the impact of these acute and chronic affections on the experience of the families of patients and on social integration.

## Abbreviations

ARCI: Autosomal Recessive Congenital Ichthyosis; CIE: Congenital Ichtyosiform Erythroderma; FBI: Family Burden Ichthyosis; LI: Lamellar Ichthyosis; MAGEC: MAladie Génétique à Expression Cutanée; QoL: Quality of Life; SF-12: Short-Form Q-12.

## Competing interests

The study was conduct in association with Eau Thermale Avène.

## Authors’ contributions

HD was involved in acquisition and interpretation of data; helped to draft the manuscript and participated in revising it critically. SHR was involved in acquisition of data and was involved in writing and revising the manuscript for important intellectual content. CM and VS were involved in revising the manuscript. CB contributed in establishing conception and design of study, and was involved in writing and revising the manuscript for important intellectual content. CT carried out basic conception and design of study, analysis and interpretation of data, was involved in analysis of data, statistical analysis and revising the manuscript. All authors read and approved the final manuscript.
